# 3D Printing of Triamcinolone Acetonide in Triblock Copolymers of Styrene–Isobutylene–Styrene as a Slow-Release System

**DOI:** 10.3390/polym14183742

**Published:** 2022-09-07

**Authors:** Philipp S. Hilgeroth, Justus F. Thümmler, Wolfgang H. Binder

**Affiliations:** Macromolecular Chemistry, Institute of Chemistry, Martin-Luther University Halle-Wittenberg, Von-Danckelmann-Platz 4, 06120 Halle, Germany

**Keywords:** SIBS triblock copolymer, Triamcinolone acetonide, 3D printing, living carbocationic polymerization

## Abstract

Additive manufacturing has a wide range of applications and has opened up new methods of drug formulation, in turn achieving attention in medicine. We prepared styrene–isobutylene–styrene triblock copolymers (SIBS; Mn = 10 kDa–25 kDa, PDI 1,3–1,6) as a drug carrier for triamcinolone acetonide (TA), further processed by fused deposition modeling to create a solid drug release system displaying improved bioavailability and applicability. Living carbocationic polymerization was used to exert control over block length and polymeric architecture. Thermorheological properties of the SIBS polymer (22.3 kDa, 38 wt % S) were adjusted to the printability of SIBS/TA mixtures (1–5% of TA), generating an effective release system effective for more than 60 days. Continuous drug release and morphological investigations were conducted to probe the influence of the 3D printing process on the drug release, enabling 3D printing as a formulation method for a slow-release system of Triamcinolone.

## 1. Introduction

The 3D printing of pharmaceuticals has become an attractive alternative to conventional formulation technologies in the past decade [1,2,3,4]. The use of various printing technologies such as fused deposition modeling (FDM), stereo lithography (SLS), or binder jet printing and laser sintering has revolutionized formulation strategies by implementing solutions for personalized medicines [5,6] or complex dosage regimes, such as polypills or dosage of medicines with a low therapeutic index [1]. A variety of different drugs have been 3D-printed using various polymers, such as poly-ethylene glycol (PEG), poly-lactic acid (PLA), poly-vinyl alcohol (PVA), poly-caprolactone (PCL), poly-isobutylene (PIB), hydroxypropylmethylcellulose (HMPC), and poly-vinylpyrrolidon (PVP)-type polymers [5,7,8,9,10,11,12,13,14,15,16,17,18,19,20,21], always considering key parameters such as thermal stability of the drug, melt flow of the polymers, as well as their final shape persistence and (bio)-degradation, as desired. Apart from the use of biodegradable polymers (such as PLA, PCL), 3D printing of non-degradable polymers for implantation and slow release over many years has gained notable importance in this context [8,11,19,20,21,22,23]. In particular, the use of poly-isobutylene (IB)-based polymers has become important as a 3D-printable medium [24,25,26], able to embed a variety of drugs (with a focus on paclitaxel^®^), especially for long-term release [27,28,29,30,31,32,33,34,35,36,37,38]. To effect sufficient processability, and, most of all, 3D-printability, copolymers of IB with styrene (S) have been designed. The triblock copolymer SIBS is known for its medical use, in e.g., the TAXUS^®^ coronary stent as a drug carrier for paclitaxel, which is a known antitumor drug/cell growth inhibitor [39,40,41]. For this application, a stent is spray-coated with a solution of drug and polymer, followed by solvent evaporation to generate a thin film containing the embedded drug. After implantation, the drug will be released over a long time period. It is reported that SIBS as a drug carrier forms a very slow-release system, releasing drugs over years [42,43]. Advantages of SIBS when compared to PLA or other biodegradable polymers include its excellent stability in the human body, where the structure can be kept over a long period of time, which leads to long-term drug exposure. Thus, synthetic heart valves have been designed using SIBS with low styrene content, a polyester fabric, and a second SIBS with higher styrene content for structural stability [36,44]. The mechanical properties, in particular, are important in such SIBS polymers, as they can be engineered relatively easily, by adjusting the ratios of hard and soft segments. For medical applications, these polymers are usually designed with a molecular weight in the range of 75–150 kDa [45]. To increase the mechanical properties, it is common to blend SIBS with thermoplastic polymers such as PS or PPO, which, due to their improved thermoplastic properties, can be used for additive manufacturing using FFF 3D printing [46].

Triamcinolone acetonide (TA) is known for its excellent thermal stability at up to 290 °C [47] and is commonly used to treat skin and joint conditions as well as ocular diseases [48]. The drug is conventionally applied as a topical medication or as an injection, and for a less troublesome treatment, a long-term delivery system would be advantageous. Formulations of TA using a polymer matrix are usually fast-release systems using biodegradable polymers such as PEG [49,50], PLA [51], or PCl [52,53]. With a strong burst release in the first hour, the drug content of the subsequent release is significantly smaller. A release system of the penta-block copolymer PLA-PCl-PEG-PCl-PLA was reported, showing full release after 40 days, depending on the length of hydrophobic segments inside the polymer backbone [54].

**Figure 1:**
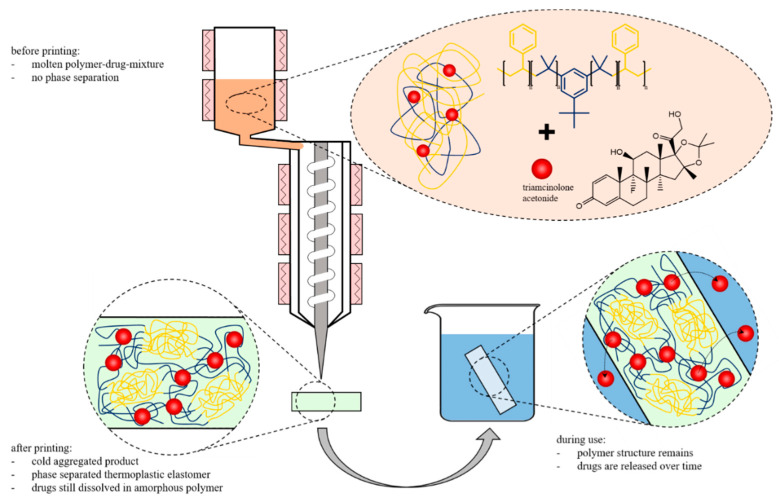
Concept of the 3D printing of triamcinolone acetonide (TA, red) embedded into SIBS triblock copolymers (S = yellow, IB = blue).

The use of a 3D-printable hydrophobic polymer such as SIBS, known as a slow-release system with adjustable properties, could lead to a new area of application of triamcinolone acetonide. Using 3D printing, various shapes can be designed, which opens up further applications. Here, we report studies on the 3D printing of SIBS/triamcinolone acetonide (TA) blends, based on the rheological properties of synthesized low-molecular-weight SIBS triblock copolymers containing TA, with a particular focus on shear rate dependence and the effect of higher printing temperatures on the release system (see Figure 1). 

## 2. Materials and Methods

Triamcinolone acetonide (4aS,4bR,5S,6aS,6bS,9aR,10aS,10bS)-4b-fluoro-6b-glycoloyl-5-hydroxy-4a,6a,8,8-tetramethyl-4a,4b,5,6,6a,6b,9a,10,10a,10b,11,12-dodecahydro-2H-naphtho [2′,1′:4,5]indeno[1,2-d][1,3]dioxol-2-one) was purchased from ABCR (Karlsruhe, Germany). Sodium chloride was obtained from Roth (Dessau, Germany). Na_2_HSO_4_ was purchased from Alfa Aesar ((Haverhill, MA, USA). KH_2_SO_4_ was purchased from Roanal (Budapest, Hungary). Potassium chloride was purchased from Sigma-Aldrich (St. Louis, MO, USA). All substances used were of analytical grade or higher and used without further purification if not stated otherwise. Double-distilled water was used in the experiments. Solvent handling and the chemicals that are not mentioned here can be found in the Supplementary Materials.

All ^1^H-NMR and ^13^C-NMR spectra were measured with a Varian FT-NMR spectrometer (500 and 101 MHz, respectively, Agilent Technologies, Waldbronn, Germany). The samples were measured at 27 °C using deuterated chloroform (CDCl_3_). All NMR spectral analysis was performed using MestReNova (Version: 12.0.2-20910, Mestrelab Research, Santiago de Compostela, Spain).

The synthesized polymer was analyzed via gel permeation chromatography (GPC) using a Viscothek GPCmax VE2001 (ViscoTec, Tönning a. Inn, Germany), equipped with two columns (CLM-3008, CLM-3011) and a refractive index (RI) detector (Viscothek 3580). Linear poly-isobutylene (PIB) was used for standard calibration, and THF (HPLC grade, Prolabo) was used as an elution solvent at a flow rate of 1 mL/min. The injection concentration was between 4 mg/mL and 5 mg/mL.

Differential scanning calorimetry (DSC) (Netzsch DSC 204 F1 Phoenix, Netzsch, Selb, Germany) was used to analyze the thermal properties of the synthesized polymers. The sample (10 mg) was heated from −100 °C to 200 °C, using a heating rate of 10 K/min and a nitrogen atmosphere. The data were collected in the second heating cycle. Nitrogen (20 mL/min) was used as an inert gas. Data analysis was performed on the software NETZSCH Proteus (version 5.2.1, Netzsch, Selb, Germany) and Origin 2019 (OriginLab Corporation, Northampton, MA, USA).

Thermogravimetric analysis (TGA) was performed using a TG 209 F3 Tarus by Netzsch. Samples (15 mg) were weighed in an aluminum oxide crucible and heated under nitrogen atmosphere at a heating rate of 10 K/min until a temperature of 600 °C was reached. For data analysis, the software Netzsch Proteus for Thermal Analysis (Version 5.2.1, Netzsch, Selb, Germany) was used.

Rheology experiments were performed with an MCR 101-DSO (Anton Paar, Graz, Austria) using a parallel plate geometry (diameter 8 mm). The measurements were performed from 140 °C to 180 °C with 20 min of equilibration time in between the measurements. The shear rate was set in the range of 0.1–100 s^−1^. Data analysis was performed with the software RheoCompass^TM^ (version V1.30.1064, Anton Paar, Graz, Austria) and Origin 2019.

For quantification of triamcinolone acetonide, high-performance liquid chromatography (HPLC) was used. Experiments were conducted on an Atlantis T3 5 µm (4.6 × 250 mm) column. ACN, water, and formic acid (60:40:0.1) were used as a solvent. Solutions of the two drugs in methanol in a range of 0.001–0.2 mg/mL were used to obtain a calibration curve (Figure S9). To determine the total drug load, samples were dissolved in 1 mL THF. Methanol (4 mL) was added to precipitate the SIBS polymer and the sample was then filtered using a 0.2 µm PTFE filter to remove the precipitate.

For 3D printing, a 3D Discovery G5 from REGENHU was used. The printer was equipped with a thermo polymer extruder type HM-300H (REGENHU, Villaz-Saint-Pierre, Switzerland) using a 0.33 mm diameter needle. The printing was carried out using a pressure of 0.4 MPa with a feed of 10 rev s/mm. The printing temperatures were 180 °C and 200 °C for the drug-containing polymer and pure SIBS, respectively. The temperature of the storage tank was set to a 10 °C higher temperature.

AFM measurements were performed using a Nanosurf CoreAFM (Nanosurf, Liestal, Switzerland) with Tap190AI-G Cantilevers in the phase contrast mode. The samples (10 mg) were dissolved in DCM, dropped onto the sample carrier, and left at room temperature to evaporate, followed by high vacuum for 24 h to ensure no solvent residue. Data analysis was accomplished using Gwyddion 2.61 (Czech Metrology Institute, Brno, Czech Republic).

The release of triamcinolone acetonide was studied in phosphate-buffered saline (PBS), pH 7.4. The extruded and film samples (10–25 mg) were weighed in the buffer solution to completely submerge the polymer/drug sample. Sink conditions were ensured during the whole release experiment, using 50 mL of solvent. The experiment was performed at 37 °C ambient temperature and shaking at 150 min^−1^. Samples of 0.5 mL were taken in previously described time intervals and analyzed via HPLC as mentioned before. Drug release was calculated from TA in the release medium, including a correction function for the 1% solvent removal after each sample was taken.

## 3. Results and Discussion

### 3.1. Synthesis

**Scheme 1:**
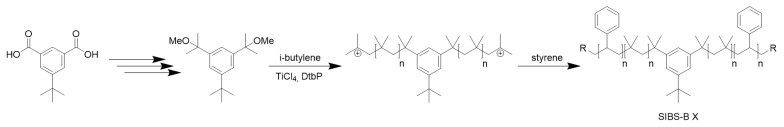
Synthesis of SIBS polymers starting from 5-(tert-butyl)isophthalic acid to prepare the bivalent initiator 1-(tert-butyl)-3,5-bis(2-methoxypropan-2-yl)benzene, followed by living carbo cationic polymerization (LCCP) of iso-butylene (IB), followed by addition of styrene (S). Indicated carbocations are representative of the active (growing) chain ends (reversibly deactivated chains are not shown).

Several triblock copolymers with adjustable composition of the blocks (S, IB) were synthesized via living carbo cationic polymerization (LCCP) using a bivalent initiator (Figures S1–S6) [55,56,57]. LCCP is known to allow for a living polymerization of both monomers, IB and S, to generate an adjustable and well-engineered block length of both of the monomers [58,59]. Within the final SIBS polymer, the S and IB blocks are microphase-segregated, imparting favorable rheological properties for this thermoplastic elastomer, with IB constituting the liquid phase and S the solid phase. To this end, LCCP was initiated by 1-(tert-butyl)-3,5-bis(2-methoxypropan-2-yl)benzene, dissolved in a mixture of hexane and DCM 60:40 at −80 °C with di-tert-butyl-pyridine (DtbP) as the acid scavenger. Isobutylene (IB) was added and the polymerization was initiated with the addition of TiCl_4_. After 10 min, styrene (S) was added at −80 °C to effect the crossover chemistry from the living PIB chains to the S-polymer, subsequently quenched using methanol after 15 min (Scheme 1). The polymer was precipitated into a 10-fold excess of methanol and dried using high vacuum (0.007 mbar). A series of different SIBS triblock copolymers displaying molecular weights from 10 kDa to 25 kDa, with block ratios ranging from IB/S = 20:1 to 1:1, were prepared (see Table 1) with reasonable polydispersities and with controlled molecular weights (Figures S7 and S8).

After testing with rheology, **SIBS-B4** (vide supra) was chosen due to its excellent properties, also displaying an acceptable PS/PI ratio and an acceptable PDI. The synthesized polymers with their PDI and molecular weights are shown in Table 1. Polymer and drug mixtures were prepared by dissolving both in THF and removing the solvent under reduced pressure. The SIBS drug mixtures were then dried using a high-vacuum pump to ensure removal of all solvent residues.

### 3.2. Thermal Analysis

**Figure 2:**
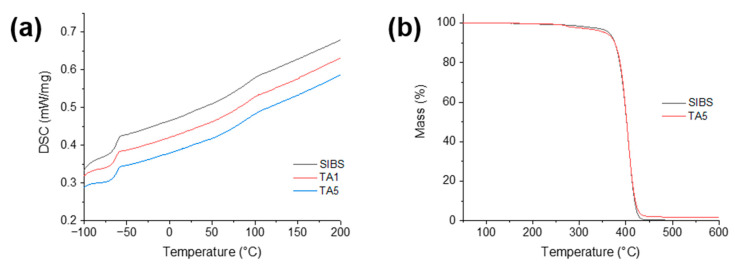
(**a**) Differential scanning calorimetry (DSC) measurements of SIBS and formulations with TA at different weight percentages (1 wt % and 5 wt %). (**b**) Thermogravimetric analysis (TGA) measurements of SIBS and its formulations with the drug.

To guarantee successful printability during the melt extrusion process in 3D printing, we probed the thermal stability of the polymer and the SIBS–drug mixtures. Differential scanning calorimetry (DSC) was conducted to understand the influence of the drug on the T_g_-values of the polymer. Thermal gravimetric analysis (TGA) was accomplished to ensure that no thermal degradation was taking place during the 3D printing process, and that neither the SIBS nor the drug are decomposing at the respective printing temperature. The corresponding graphs of SIBS-B4 and its mixtures with 1–5 wt % TA are shown in Figure 2. TGA showed thermal stability up to 250 °C, which is higher than the expected printing temperature. DSC shows the presence of two glass transition temperatures, one T_g_ at −60 °C, which can be assigned to the soft PIB segments, and the second one at 70 °C, indicative of the PS block, overall proving microphase segregation of the two polymer blocks. The addition of the drug in amounts of 1–5 wt % of TA shows no significant change in the thermal properties, proving that the properties of the SIBS polymer are largely unchanged.

### 3.3. Rheology

For 3D printing, the viscosity dependent on the shear rate and the temperature is the most important factor to ensure adequate extrusion of the polymer. The shear rate at the nozzle and the polymer tank leads to the so-called “printing window”, in which the polymer matches the requirements of the polymer extruder. For our printing system, the viscosity of the polymer must be in the range of 200 to 2000 Pa·s to be extruded in a controlled manner, excluding primordial dripping from the printing head as well as a successful extrusion. Given the data collected in the DSC measurement, the temperature for 3D printing should be above 100 °C to ensure a homogeneous state throughout the mixture.

**Figure 3:**
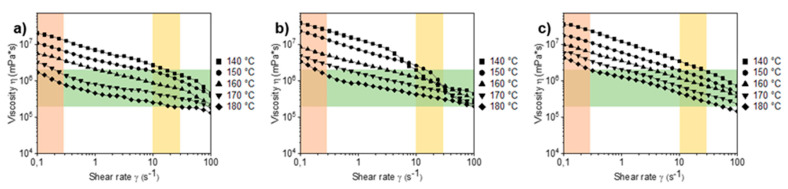
Rheology measurements of (**a**) SIBS-B4; (**b**) SIBS-B4 mixed with TA (1 wt %); (**c**) SIBS-B4 mixed with TA (5 wt %) displaying viscosity η vs. shear rate γ measured at different temperatures.

The conducted rheology measurements underscore the 3D-printability of all samples with viscosities of 150 Pa·s up to 40 kPa·s for the different samples (Figure 3). The results also showed that the addition of the drug to the polymer led to an increase in viscosity. At a temperature of 140 °C, both drug-containing samples (1 wt % and 5 wt % TA) displayed a viscosity of 35 kPa·s, compared to 20 k kPa·s of the virgin sample at low shear rates. With increasing temperature, this ratio remained constant, with 3.4 kPa·s and 4.1 kPa·s for 1 wt % and 5 wt %, respectively, compared to 1.7 kPa·s of pure SIBS-B4. Thus, all of the samples showed suitable viscosities for 3D printing at different temperatures. At a shear rate of 10 s^−1^, the virgin sample displayed a viscosity of 253 Pa·s, and the drug-loaded samples showed viscosities of 420 kPa·s and 460 kPa·s for 1 wt % and 5 wt %, respectively. For SIBS-B4, the viscosity is matching starting from a temperature of 170 °C, where the decreasing viscosity of the drug-loaded samples with higher temperatures indicated that the mixtures are displaying suitable viscosities for the polymer tank from temperatures of 190 °C and above.

### 3.4. 3D Printing

**Figure 4:**
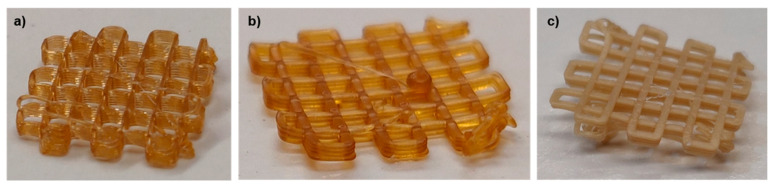
Photos of 3D-printed grids of 10 mm × 10 mm. (**a**) SIBS-B15; (**b**) SIBS-B15 mixed with TA (5 wt %); (**c**) SIBS-B15 mixed with TA (5 wt %) after the drug release. Height of the printed specimen is approx. 5 mm.

SIBS-B4 and the polymer–drug mixtures with 1 wt % and 5% TA, respectively, were printed successfully. The printing parameters are shown in Table 2. As reported in the literature, 3D printing using FDM of high-molecular-weight SIBS is not possible [46,60]; thus, our synthesized medium-to-low-molecular-weight SIBS-B4 was well suited for the printing process, requiring a temperature of only 210 °C for a successful controlled extrusion. The polymer–drug mixtures were extrudable at lower temperatures due to the higher applied pressure of 0.4 MPa. The final printed samples show suitable resolution and uniform strand thickness of 0.33 mm (Figure 4). The generated structures were stable and contained the drug in the indicated amount, as revealed during the subsequent release studies (see next section).

### 3.5. Atomic Force Microscopy

**Figure 5:**
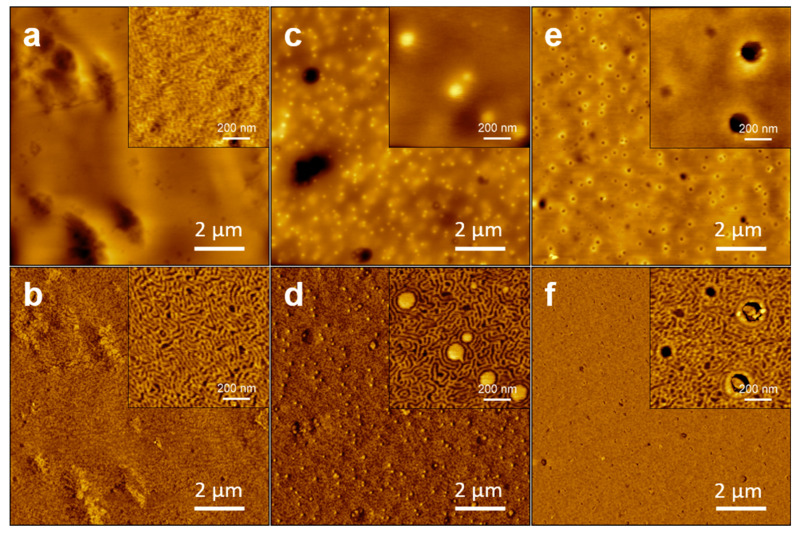
AFM images of SIBS and SIBS/TA formulations. (**a**) Height and (**b**) phase images of SIBS-B15; (**c**) height and (**d**) phase images of SIBS-B15 mixed with TA (5 wt %); (**e**) height and (**f**) phase images of SIBS-B15 mixed with TA (5 wt %) after storing in methanol for 1 h. Scale bars are 2 µm and 200 nm for the magnified insets.

To understand the micro-structure and drug incorporation into the polymer in more detail, AFM (atomic force microscopy) was performed (Figure 5). Previous morphology studies have already been conducted for SIBS and similar styrene-containing block copolymers such as SIPS [61] (IP = isoprene), SBS [62,63] (B = butadiene), and SEBS [64] (EB = ethylene/butylene), mainly displaying the formation of a partly lamellar phase separation or random structures, as proven via AFM and/or TEM [65]. Thus, the surface of these ABA-type triblock copolymers usually shows a topography that consists of valleys and hills. The hills are formed by the hard segment (styrene) and the valleys consist of the soft rubbery phase (IP/B/IB/EB). The percentage and height of the hard segments is reported to increase in size with increasing PS content [64]. The size of these phases are in the diameter of 20 nm edge to edge and 40 nm center to center, at high molecular weights from 100 kDa to 175 kDa [66].

In paclitaxel-containing stents, the drug shows a phase separation from the SIBS matrix, as it is very hydrophobic. With a Hansen solubility parameter (HSP) of 28.59 MPa^0.5^ [67], the solubility of paclitaxel shows a big difference from the involved polymers, with 16.05 MPa^0.5^ and 18.61 MPa^0.5^ for isobutylene and styrene, respectively [68]. For TA with HSP of 20.1 MPa^0.5^ [69], this drug should be more likely to dissolve in the amorphous IB segments of the matrix.

The pure SIBS-B4 polymer shows the expected unorganized phase separation (Figure 5a,b) after coating and drying the polymer. The polymer forms two phases with a medium thickness of 20 nm. The addition of the drug shows no impact on the phase separation of the SIBS, where the drug forms small crystallites with a size of 120 nm/50 nm, and therefore seems to not be fully dissolved in the polymer. In close proximity to the drug aggregates, a more controlled phase separation in the form of rings is observed (Figure 5c,d). In order to simulate a fast release, the sample was stored in methanol for 1 h, after which the AFM experiment was repeated. The AFM pictures show a removal of the drug and a persistency of the holes left behind (Figure 5e,f). The polymer still shows phase separation, which indicates no significant interactions with the solvent, neither in view of a solvent-induced swelling nor the destruction of the phase structure at all.

### 3.6. Drug Release

SIBS is known to form a slow-release system. Release studies of paclitaxel usually show a burst release in the first days of study, after which the drug is released over a long period of time at a nearly constant rate, independent of the drug load [70]. With a higher paclitaxel fraction, this release rate is increasing, often explained by the phase segregation between the S and IB blocks. It also has been reported that the conditions of preparation can influence the release from a stent, as well [71]. For our system, we compare the release of a film to the 3D-printed samples to show the influence of shear rate and high temperature on the system.

**Figure 6:**
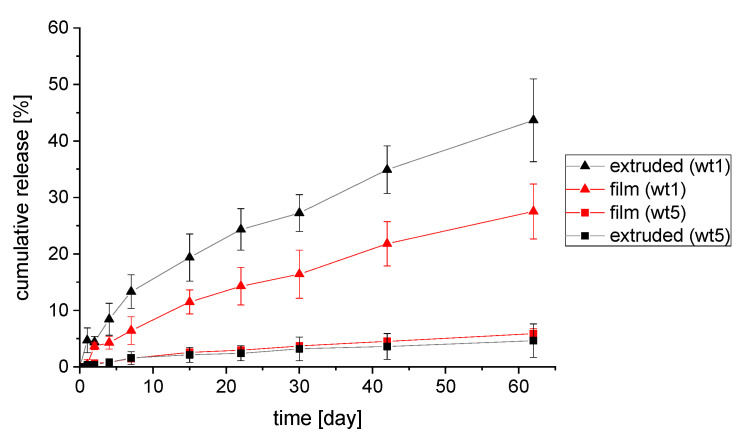
Drug release study of TA from the SIBS matrix.

The release studies (Figure 6) show similar release kinetics, known for paclitaxel release, and no major influence of the extrusion on the release, with a burst release in the first 7 days of the release study and a nearly linear lease behavior for the following weeks up to 60 days. There also is a faster cumulative release for the less-loaded samples (1 wt % TA) with a total of 43% and 27% release for the extruded samples and the prepared films, respectively. The samples containing more of the drug (5 wt %) also show a burst release in the first 7 days, followed by an almost linear release kinetic for the following weeks. With a higher drug load, however, the cumulative release is smaller. We hypothesize a significant influence of the surface area on the release kinetics, as the films and the extruded sample differ. We propose that initially, both sample surfaces are saturated with the drug, supported by the ratios of cumulative release, compared between samples of the same preparation method. The initial films show a release ratio of 5.8% to 27.5%, which well matches the ratio of 1:5 of the initial drug load. For the extruded samples, the samples show a cumulative release of 4.7% to 43%. We propose that during extrusion, there is a significant influence of shear stress and temperature on the phase segregation of drug and matrix and thus the drug distribution in the polymer, especially at the surface. With higher drug loads, the interactions between matrix and drug could result in a saturated surface and an even distribution in the polymer, whereas a lower drug load would form a gradient from a saturated surface and higher concentration in the outer regions of the polymer strand.

## 4. Conclusions

We investigated the impact of 3D printing using FDM on the release of triamcinolone acetonide from a SIBS triblock copolymer matrix. Low-molecular-weight (10–25 kDa) SIBS triblock copolymers were synthesized using carbocationic polymerization with a bivalent initiator, reaching controllable molecular weights and reasonably low polydispersities. To determine thermal stability and thermoplastic elastomeric properties, TGA and DSC were conducted, indicating adequate thermal stability of the drug and polymer up to 250 °C and glass transition temperatures of the PIB and PS blocks of −60 °C and 70 °C, respectively. Melt rheology was performed to determine the suitability for 3D printing, and the printing temperatures were found to be in the range of 180 °C to 200 °C, which led to a successful extrusion and 3D printing of the polymer–drug formulations. To understand the microstructure of the formulation, AFM was conducted and the characteristic phase separation between the IB and the S block in SIBS was visible. The pictures also indicate phase separation of the polymer and the drug, forming small circular crystallites with ~150 nm diameter, which, after a simulated release of the drugs, leaves holes in the surface of the previous position of the drug. A detailed time-dependent drug release study was conducted over a time period of 60 days to investigate the influence of shear force and temperature applied to the formulation during the 3D printing extrusion process. All samples showed a burst release in the first 7 days and a slow and steady release in the following period. The studies indicate a correlation between cumulative drug release and surface area independent of the drug load, assuming the presence of a saturated surface. At its core, this work demonstrates the use of SIBS as an extrudable and 3D printable carrier for triamcinolone acetonide, necessitating further investigations of the detailed microstructure of the SIBS polymer, along with the exact location of the drug in specific parts of the phase.

## Figures and Tables

**Table 1:** Synthesized SIBS triblock copolymers with various molecular weights (Mn), polydispersities (PDI), and the calculated wt % of the PS block.

Sample (SIBS)	Mn (GPC)	PDI	wt % PS
B1	10,800	1.36	6%
B2	14,000	1.8	32%
B3	18,000	1.47	36%
**B4**	**22,300**	**1.44**	**38%**
B5	25,200	1.65	49%

**Table 2:** FDM printing parameters of polymer SIBS-B4, containing 1 wt % (TA1) and 5 wt % (TA5) of triamcinolone acetonide (TA), respectively.

Sample	T_Tank_	T_Extruder_	Pressure (MPa)	Feed (mm·s ^−1^)
SIBS-B4	210	190	0.2	10
TA1	190	180	0.4	10
TA5	190	180	0.4	10

## Data Availability

Data are contained within the article.

## Supplementary Materials

The following supporting information can be downloaded at: https://www.mdpi.com/article/10.3390/polym14183742/s1. Figure S1: ^1^H-NMR spectrum of dimethyl-5-(tert-butyl)isophthalate (1). Figure S2: ^13^C-NMR spectrum of dimethyl-5-(tert-butyl)isophthalate (1). Figure S3: ^1^H-NMR spectrum of 2,2‘-(5-(tert-butyl)-1,3-phenylene)bis(propan-2-ol) (2). Figure S4: ^13^C-NMR spectrum of 2,2‘-(5-(tert-butyl)-1,3-phenylene)bis(propan-2-ol) (2). Figure S5: ^1^H-NMR spectrum of 1-(tert-butyl)-3,5-bis(2-methoxypropan-2-yl)benzene (3). Figure S6: ^13^C-NMR spectrum of 1-(tert-butyl)-3,5-bis(2-methoxypropan-2-yl)benzene (3). Figure S7: ^1^H-NMR spectrum of SIBS-B4. Figure S8: GPC graphs of all synthesized SIBS-polymers. Figure S9: HPLC calibration for TA from 0.001 mg/mL to 0.2 mg/mL.

## References

[1] Trenfield S.J., Awad A., Goyanes A., Gaisford S., Basit A.W. (2018). 3D Printing Pharmaceuticals: Drug Development to Frontline Care. Trends Pharmacol. Sci..

[2] Awad A., Trenfield S.J., Goyanes A., Gaisford S., Basit A.W. (2018). Reshaping drug development using 3D printing. Drug Discov. Today.

[3] Alhnan M.A., Okwuosa T.C., Sadia M., Wan K.W., Ahmed W., Arafat B. (2016). Emergence of 3D Printed Dosage Forms: Opportunities and Challenges. Pharm. Res..

[4] Goole J., Amighi K. (2016). 3D printing in pharmaceutics: A new tool for designing customized drug delivery systems. Int. J. Pharm..

[5] Zema L., Melocchi A., Maroni A., Gazzaniga A. (2017). Three-Dimensional Printing of Medicinal Products and the Challenge of Personalized Therapy. J. Pharm. Sci..

[6] Alomari M., Mohamed F.H., Basit A.W., Gaisford S. (2015). Personalised dosing: Printing a dose of one’s own medicine. Int. J. Pharm..

[7] Smith D.M., Kapoor Y., Klinzing G.R., Procopio A.T. (2018). Pharmaceutical 3D printing: Design and qualification of a single step print and fill capsule. Int. J. Pharm..

[8] Luzuriaga M.A., Berry D.R., Reagan J.C., Smaldone R.A., Gassensmith J.J. (2018). Biodegradable 3D printed polymer microneedles for transdermal drug delivery. Lab Chip.

[9] Kollamaram G., Croker D.M., Walker G.M., Goyanes A., Basit A.W., Gaisford S. (2018). Low temperature fused deposition modeling (FDM) 3D printing of thermolabile drugs. Int. J. Pharm..

[10] Kempin W., Domsta V., Grathoff G., Brecht I., Semmling B., Tillmann S., Weitschies W., Seidlitz A. (2018). Immediate Release 3D-Printed Tablets Produced Via Fused Deposition Modeling of a Thermo-Sensitive Drug. Pharm. Res..

[11] Kadry H., Al-Hilal T.A., Keshavarz A., Alam F., Xu C., Joy A., Ahsan F. (2018). Multi-purposable filaments of HPMC for 3D printing of medications with tailored drug release and timed-absorption. Int. J. Pharm..

[12] Fu J., Yu X., Jin Y. (2018). 3D printing of vaginal rings with personalized shapes for controlled release of progesterone. Int. J. Pharm..

[13] Ehtezazi T., Algellay M., Islam Y., Roberts M., Dempster N.M., Sarker S.D. (2018). The Application of 3D Printing in the Formulation of Multilayered Fast Dissolving Oral Films. J. Pharm. Sci..

[14] Maroni A., Melocchi A., Parietti F., Foppoli A., Zema L., Gazzaniga A. (2017). 3D printed multi-compartment capsular devices for two-pulse oral drug delivery. J. Control. Release.

[15] Genina N., Boetker J.P., Colombo S., Harmankaya N., Rantanen J., Bohr A. (2017). Anti-tuberculosis drug combination for controlled oral delivery using 3D printed compartmental dosage forms: From drug product design to in vivo testing. J. Control. Release.

[16] Beck R.C.R., Chaves P.S., Goyanes A., Vukosavljevic B., Buanz A., Windbergs M., Basit A.W., Gaisford S. (2017). 3D printed tablets loaded with polymeric nanocapsules: An innovative approach to produce customized drug delivery systems. Int. J. Pharm..

[17] Holländer J., Genina N., Jukarainen H., Khajeheian M., Rosling A., Mäkilä E., Sandler N. (2016). Three-dimensional printed PCL-based implantable prototypes of medical devices for controlled drug delivery. J. Pharm. Sci..

[18] Goyanes A., Kobayashi M., Martinez-Pacheco R., Gaisford S., Basit A.W. (2016). Fused-filament 3D printing of drug products: Microstructure analysis and drug release characteristics of PVA-based caplets. Int. J. Pharm..

[19] Goyanes A., Det-Amornrat U., Wang J., Basit A.W., Gaisford S. (2016). 3D scanning and 3D printing as innovative technologies for fabricating personalized topical drug delivery systems. J. Control. Release.

[20] Weisman J.A., Nicholson J.C., Tappa K., Jammalamadaka U., Wilson C.G., Mills D.K. (2015). Antibiotic and chemotherapeutic enhanced three-dimensional printer filaments and constructs for biomedical applications. Int. J. Nanomed..

[21] Melocchi A., Parietti F., Loreti G., Maroni A., Gazzaniga A., Zema L. (2015). 3D printing by fused deposition modeling (FDM) of a swellable/erodible capsular device for oral pulsatile release of drugs. J. Drug. Deliv. Sci. Technol..

[22] Suaste-Gómez E., Rodríguez-Roldán G., Reyes-Cruz H., Terán-Jiménez O. (2016). Developing an ear prosthesis fabricated in polyvinylidene fluoride by a 3D printer with sensory intrinsic properties of pressure and temperature. Sensors.

[23] Goyanes A., Buanz A.B., Hatton G.B., Gaisford S., Basit A.W. (2015). 3D printing of modified-release aminosalicylate (4-ASA and 5-ASA) tablets. Eur. J. Pharm. Biopharm..

[24] Rupp H., Döhler D., Hilgeroth P., Mahmood N., Beiner M., Binder W.H. (2019). 3D Printing of Supramolecular Polymers: Impact of Nanoparticles and Phase Separation on Printability. Macromol. Rapid. Commun..

[25] Rupp H., Binder W.H. (2020). 3D Printing of Core–Shell Capsule Composites for Post—Reactive and Damage Sensing Applications. Adv. Mater. Technol..

[26] Rupp H., Binder W.H. (2021). Multicomponent Stress-Sensing Composites Fabricated by 3D-Printing Methodologies. Macromol. Rapid. Commun..

[27] Wu Y.B., Li K., Xiang D., Zhang M., Yang D., Zhang J.H., Mao J., Wang H., Guo W.L. (2018). Surface immobilization of heparin on functional polyisobutylene-based thermoplastic elastomer as a potential artificial vascular graft. Appl. Surf. Sci..

[28] Jindal A., Puskas J.E., McClain A., Nedic K., Luebbers M.T., Baker J.R., dos Santo B.P., Camassola M., Jennings W., Einsporn R.L. (2018). Encapsulation and release of Zafirlukast from electrospun polyisobutylene-based thermoplastic elastomeric fiber mat. Eur. Polym. J..

[29] Trant J.F., McEachran M.J., Sran I., Turowec B.A., de Bruyn J.R., Gillies E.R. (2015). Covalent Polyisobutylene-Paclitaxel Conjugates for Controlled Release from Potential Vascular Stent Coatings. ACS Appl. Mater. Interfaces.

[30] Trant J.F., Sran I., de Bruyn J.R., Ingratta M., Borecki A., Gillies E.R. (2015). Synthesis and properties of arborescent polyisobutylene derivatives and a paclitaxel conjugate: Towards stent coatings with prolonged drug release. Eur. Polym. J..

[31] Trant J.F., Abd Rabo Moustafa M.M., Sran I., Gillies E.R. (2016). Polyisobutylene—Paclitaxel conjugates with pendant carboxylic acids and polystyrene chains: Towards multifunctional stent coatings with slow drug release. J. Polym. Sci. Part A Polym. Chem..

[32] Schulz M., Binder W.H. (2015). Mixed Hybrid Lipid/Polymer Vesicles as a Novel Membrane Platform. Macromol. Rapid. Commun..

[33] Ren K., Zhang M., He J., Wu Y., Ni P. (2015). Preparation of Polymeric Prodrug Paclitaxel-Poly(lactic acid)-b-Polyisobutylene and Its Application in Coatings of a Drug Eluting Stent. ACS Appl. Mater. Interfaces.

[34] Schulz M., Werner S., Bacia K., Binder W.H. (2013). Controlling molecular recognition with lipid/polymer domains in vesicle membranes. Angew. Chem. Int. Edit..

[35] Ellis S.G., Stone G.W., Cox D.A., Hermiller J., O’Shaughnessy C., Mann T., Turco M., Caputo R., Bergin P.J., Bowman T.S. (2009). Long-term safety and efficacy with paclitaxel-eluting stents: 5-year final results of the TAXUS IV clinical trial (TAXUS IV-SR: Treatment of De Novo Coronary Disease Using a Single Paclitaxel-Eluting Stent). JACC Cardiovasc. Interv..

[36] Pinchuk L., Wilson G.J., Barry J.J., Schoephoerster R.T., Parel J.M., Kennedy J.P. (2008). Medical applications of poly(styrene-block-isobutylene-block-styrene) (“SIBS”). Biomaterials.

[37] Puskas J.E., Chen Y. (2004). Biomedical application of commercial polymers and novel polyisobutylene-based thermoplastic elastomers for soft tissue replacement. Biomacromolecules.

[38] Kennedy J.P., Rosenthal K.S., Kashibhatla B. (2004). Two generations of synthetic membranes for biological/medical applications. Des. Monomers Polym..

[39] Singla A.K., Garg A., Aggarwal D. (2002). Paclitaxel and its formulations. Int. J. Pharm..

[40] Alqahtani F.Y., Aleanizy F.S., El Tahir E., Alkahtani H.M., AlQuadeib B.T. (2019). Chapter Three—Paclitaxel. Profiles of Drug Substances, Excipients and Related Methodology.

[41] Danhier F., Lecouturier N., Vroman B., Jérôme C., Marchand-Brynaert J., Feron O., Préat V. (2009). Paclitaxel-loaded PEGylated PLGA-based nanoparticles: In vitro and in vivo evaluation. J. Control. Release.

[42] Stone G.W., Ellis S.G., Cox D.A., Hermiller J., O’Shaughnessy C., Mann J.T., Turco M., Caputo R., Bergin P., Greenberg J. (2004). A polymer-based, paclitaxel-eluting stent in patients with coronary artery disease. N. Engl. J. Med..

[43] Stone G.W., Rizvi A., Newman W., Mastali K., Wang J.C., Caputo R., Doostzadeh J., Cao S., Simonton C.A., Sudhir K. (2010). Everolimus-eluting versus paclitaxel-eluting stents in coronary artery disease. N. Engl. J. Med..

[44] Ovcharenko E., Rezvova M., Nikishau P., Kostjuk S., Glushkova T., Antonova L., Trebushat D., Akentieva T., Shishkova D., Krivikina E. (2019). Polyisobutylene-Based Thermoplastic Elastomers for Manufacturing Polymeric Heart Valve Leaflets: In Vitro and In Vivo Results. Appl. Sci..

[45] Parker T., Dave V., Falotico R. (2010). Polymers for drug eluting stents. Curr. Pharm. Des..

[46] Shen N.F., Liu S., Kasbe P., Khabaz F., Kennedy J.P., Xu W.N. (2021). Macromolecular Engineering and Additive Manufacturing of Poly(styrene-b-isobutylene-b-styrene). ACS Appl. Polym. Mater..

[47] Dandamudi M., McLoughlin P., Behl G., Rani S., Coffey L., Chauhan A., Kent D., Fitzhenry L. (2021). Chitosan-Coated PLGA Nanoparticles Encapsulating Triamcinolone Acetonide as a Potential Candidate for Sustained Ocular Drug Delivery. Pharmaceutics.

[48] Pinchuk L., Riss I., Batlle J.F., Kato Y.P., Martin J.B., Arrieta E., Palmberg P., Parrish R.K., Weber B.A., Kwon Y. (2017). The development of a micro-shunt made from poly(styrene-block-isobutylene-block-styrene) to treat glaucoma. J. Biomed. Mater. Res. B Appl. Biomater..

[49] Zgola-Grzeskowiak A., Grzeskowiak T., Zembrzuska J., Lukaszewski Z. (2006). Comparison of biodegradation of poly(ethylene glycol)s and poly(propylene glycol)s. Chemosphere.

[50] Formica M.L., Ullio Gamboa G.V., Tartara L.I., Luna J.D., Benoit J.P., Palma S.D. (2020). Triamcinolone acetonide-loaded lipid nanocapsules for ophthalmic applications. Int. J. Pharm..

[51] Doty A.C., Zhang Y., Weinstein D.G., Wang Y., Choi S., Qu W., Mittal S., Schwendeman S.P. (2017). Mechanistic analysis of triamcinolone acetonide release from PLGA microspheres as a function of varying in vitro release conditions. Eur. J. Pharm. Biopharm..

[52] Sun S., Li J., Li X., Lan B., Zhou S., Meng Y., Cheng L. (2016). Episcleral drug film for better-targeted ocular drug delivery and controlled release using multilayered poly-ε-caprolactone (PCL). Acta Biomater..

[53] Meng Y., Sun S., Li J., Nan K., Lan B., Jin Y., Chen H., Cheng L. (2014). Sustained release of triamcinolone acetonide from an episcleral plaque of multilayered poly-ε-caprolactone matrix. Acta Biomater..

[54] Tamboli V., Mishra G.P., Mitra A.K. (2013). Novel pentablock copolymer (PLA-PCL-PEG-PCL-PLA) based nanoparticles for controlled drug delivery: Effect of copolymer compositions on the crystallinity of copolymers and in vitro drug release profile from nanoparticles. Colloid Polym. Sci..

[55] Herbst F., Seiffert S., Binder W.H. (2012). Dynamic supramolecular poly(isobutylene)s for self-healing materials. Polym. Chem..

[56] Gyor M., Wang H.C., Faust R. (1992). Living Carbocationic Polymerization of Isobutylene with Blocked Bifunctional Initiators in the Presence of Di-Tert-Butylpyridine as a Proton Trap. J. Macromol. Sci. A.

[57] Orszagh I., Nagy A., Kennedy J. (1995). Living carbocationic copolymerizations. I. Synthesis and characterization of isobutylene/p‐methylstyrene copolymers. J. Phys. Org. Chem..

[58] Majoros I., Nagy A., Kennedy J. (1994). Conventional and living carbocationic polymerizations united. I. A comprehensive model and new diagnostic method to probe the mechanism of homopolymerizations. Theories and Mechanism of Phase Transitions, Heterophase Polymerizations, Homopolymerization, Addition Polymerization.

[59] Kennedy J.P. (1990). Recent developments in living carbocationic polymerization of alkenes. Macromol. Symp..

[60] Shen N., Bu J., Prevot M.E., Hegmann T., Kennedy J.P., Xu W. (2022). Macromolecular Engineering and Additive Manufacturing of Polyisobutylene-Based Thermoplastic Elastomers. II. The Poly(styrene-b-isobutylene-b-styrene)/Poly(phenylene oxide) System. Macromol. Rapid. Commun..

[61] Albert J.N.L., Young W.-S., Lewis R.L., Bogart T.D., Smith J.R., Epps T.H. (2012). Systematic Study on the Effect of Solvent Removal Rate on the Morphology of Solvent Vapor Annealed ABA Triblock Copolymer Thin Films. ACS Nano.

[62] Spontak R.J., Patel N.P. (2000). Thermoplastic elastomers: Fundamentals and applications. Curr. Opin. Colloid Interface Sci..

[63] Grady B.P., Cooper S.L., Mark J.E., Erman B., Eirich F.R. (2005). 13—Thermoplastic Elastomers. Science and Technology of Rubber.

[64] Motomatsu M., Mizutani W., Tokumoto H. (1997). Microphase domains of poly(styrene-block-ethylene/butylene-block-styrene) triblock copolymers studied by atomic force microscopy. Polymer.

[65] Knoll A., Horvat A., Lyakhova K.S., Krausch G., Sevink G.J.A., Zvelindovsky A.V., Magerle R. (2002). Phase Behavior in Thin Films of Cylinder-Forming Block Copolymers. Phys. Rev. Lett..

[66] Ranade S.V., Richard R.E., Helmus M.N. (2005). Styrenic block copolymers for biomaterial and drug delivery applications. Acta Biomater..

[67] Zhong T., Hao Y.L., Yao X., Zhang S., Duan X.C., Yin Y.F., Xu M.Q., Guo Y., Li Z.T., Zheng X.C. (2018). Effect of XlogP and Hansen Solubility Parameters on Small Molecule Modified Paclitaxel Anticancer Drug Conjugates Self-Assembled into Nanoparticles. Bioconjugate Chem..

[68] Small P.A. (1953). Some Factors Affecting the Solubility of Polymers. J. Appl. Chem..

[69] Abou-ElNour M., Ishak R.A.H., Tiboni M., Bonacucina G., Cespi M., Casettari L., Soliman M.E., Geneidi A.S. (2019). Triamcinolone acetonide-loaded PLA/PEG-PDL microparticles for effective intra-articular delivery: Synthesis, optimization, in vitro and in vivo evaluation. J. Control. Release.

[70] Ranade S.V., Miller K.M., Richard R.E., Chan A.K., Allen M.J., Helmus M.N. (2004). Physical characterization of controlled release of paclitaxel from the TAXUS^TM^ Express^2TM^ drug-eluting stent. J. Biomed. Mater. Res. A.

[71] Puskas J.E., Hoerr R.A. (2010). Drug Release from Novel Rubbery Coatings. Macromol. Symp..

