# Supplementary material for: 3D Printing of Triamcinolone Acetonide in Triblock Copolymers of Styrene–Isobutylene–Styrene as a Slow-Release System
Source: Polymers (Basel). 2022 Sep 7;14(18):3742. doi: 10.3390/polym14183742 (PMC9504042; doi:10.3390/polym14183742)
Supplement: Supplementary file 1 [file polymers-14-03742-s001.zip › 07092022polymers-1897415-supplementary. -revision corr.pdf]

Supporting Information

3D-printing of Triamcinolone Acetonide in Triblock Copolymers of Styrene-Isobutylene-Styrene as a Slow Release System

Philipp S. Hilgeroth [1], Justus F. Thümmler [1], Wolfgang H. Binder*

[1] Macromolecular Chemistry, Institute of Chemistry, Martin-Luther University Halle-Wittenberg
Von-Danckelmann-Platz 4, D-06120 Halle (Saale), Germany
* Correspondence: wolfgang.binder@chemie.uni-halle.de

Synthesis of dimethyl-5-(tert-butyl)isophthalate **(1)**[1]

5-(tert-butyl)isophthalic acid (2.5 g, 11.25 mmol) was dissolved in MeOH (100 ml). Then H_2SO_4 (1.25 ml, 23.3 mmol) was added and the reaction mixture was refluxed for 48 hours. After cooling down to room temperature, the solvent was removed under reduced pressure. The product was purified using column chromatography ($CHCl_3$) and allowed to crystallize in the freezer. After drying in vacuum, the product was obtained as white crystals with a yield of 85 % (2.4 g).

^{1}H NMR (400 MHz, $CDCl_3$): δ 8.50 (H2, t, *J* = 1.6 Hz, 1H), 8.26 (H3, d, *J* = 1.6 Hz, 2H), 3.95 (H1, s, 6H), 1.38 (H4, s, 9H).

^{13}C NMR (100 MHz, $CDCl_3$): δ 166.63, 152.13, 130.91, 130.35, 128.02, 52.27, 34.98, 31.16.

Synthesis of 2,2‘-(5-(tert-butyl)-1,3-phenylene)bis(propan-2-ol) **(2)**[1]

A 500 ml flask was heated and flushed with nitrogen thrice. Magnesium chips (4.6 g, 0.19 mmol) were dissolved in dry diethylether (100 ml) and a small portion of MeI (2 ml, 32.1 mmol) was added to the reaction. The mixture was allowed to stirr until a color change was observed. The reaction mixture was kept at 0°C, while the rest of MeI (12.5 ml, 0.2 mol) was added. In a second flask (1) (9.5 g, 37.66 mmol) was dissolved in dry diethylether (150 ml) and the solution was added to the reaction mixture over 2 hours and stirred for 16 hours. The solvent was evaporated und the crude product was recrystallized from hot ethylacetate at 80°C. After drying in vacuum, the product was obtained as white crystals with a yield of 84 % (8 g).

^{1}H NMR (400 MHz, $CDCl_3$): δ 7.43 (H3,4, m, 3H), 1.75 (H1, s, 2H), 1.60 (H2, s, 12H), 1.35 (H5, s, 9H).

^{13}C NMR (100 MHz, $CDCl_3$): δ 150.98, 148.63, 119.83, 117.65, 72.88, 35.01, 31.88, 31.50.

Synthesis of 1-(tert-butyl)-3,5-bis(2-methoxypropan-2-yl)benzene **(3)**[2]

(2) (1.9 g, 7.59 mmol) was dissolved in MeOH (30 ml). Then H_2SO_4 (0.004 ml, 0.075 mmol) was added to the reaction and the mixture was refluxed for 24 hours. After cooling down to room temperature, the solution was brought to pH 7 using $NaHCO_3$, followed by extraction with hexane (50 ml) twice. The organic phase was washed with distilled water (50 ml) four times, then dried with Na_2SO_4. The solvent was removed under reduced pressure. After drying in vacuum, the product was obtained as white crystals with a yield of 80 % (1.5 g).

^{1}H NMR (400 MHz, $CDCl_3$): δ 7.32 (H4, d, J = 1.7 Hz, 2H), 7.23 (H3, t, J = 1.7 Hz, 1H), 3.07 (H1, s, 6H), 1.54 (H2, s, 12H), 1.33 (H5, s, 9H).

^{13}C NMR (100 MHz, $CDCl_3$): δ 150.76, 145.20, 121.19, 120.35, 77.24, 50.56, 34.84, 31.51, 28.13.

Synthesis of SIBS

All solvents were dried and hexane was treated with H_2SO_4 and $H_2S_2O_7$ to remove olefins. Styrene was distilled from CaH_2 two times under reduced pressure.

A 250 ml flask was heated and flushed with nitrogen thrice. A stock solution of DCM (1 ml), DtbP (146 µl, 0.55 mmol) and (3) (61.3 mg, 0.22 mmol) were prepared in a separate flask. Hexane and DCM (55 ml; 60:40) and the stock solution were added to the reaction flask via septum and cooled down to -80°C. Iso-butylen (4.25 ml, 44.5 mmol) was condensed at -70°C in a separate flask and added to the reaction flask via septum. $TiCl_4$ (400 µl, 3.65 mmol) was added and the mixture was stirred for 10 min. A second stock solution of Hexane and DCM (12.5 ml; 60:40), DtbP (146 µl; 0.55 mmol) and styrene (4.5 ml; 39.3 mmol) was prepared in a separate flask and added to the reaction mixture. The mixture was stirred for 5 min and quenched with methanol (10 ml) and stirred for 15 min. The solvent was removed under reduced pressure, the crude polymer was dissolved in hexane and precipitated in methanol (400 ml). After drying in high vacuum, the product was obtained as a white polymer with a yield of 72 % (4.9 g). Ratio of the SI/IB blocks was calculated by integration of the NMR resonances of S (~ 7ppm) vs. IB-resonances (1,0 – 1,5 ppm).

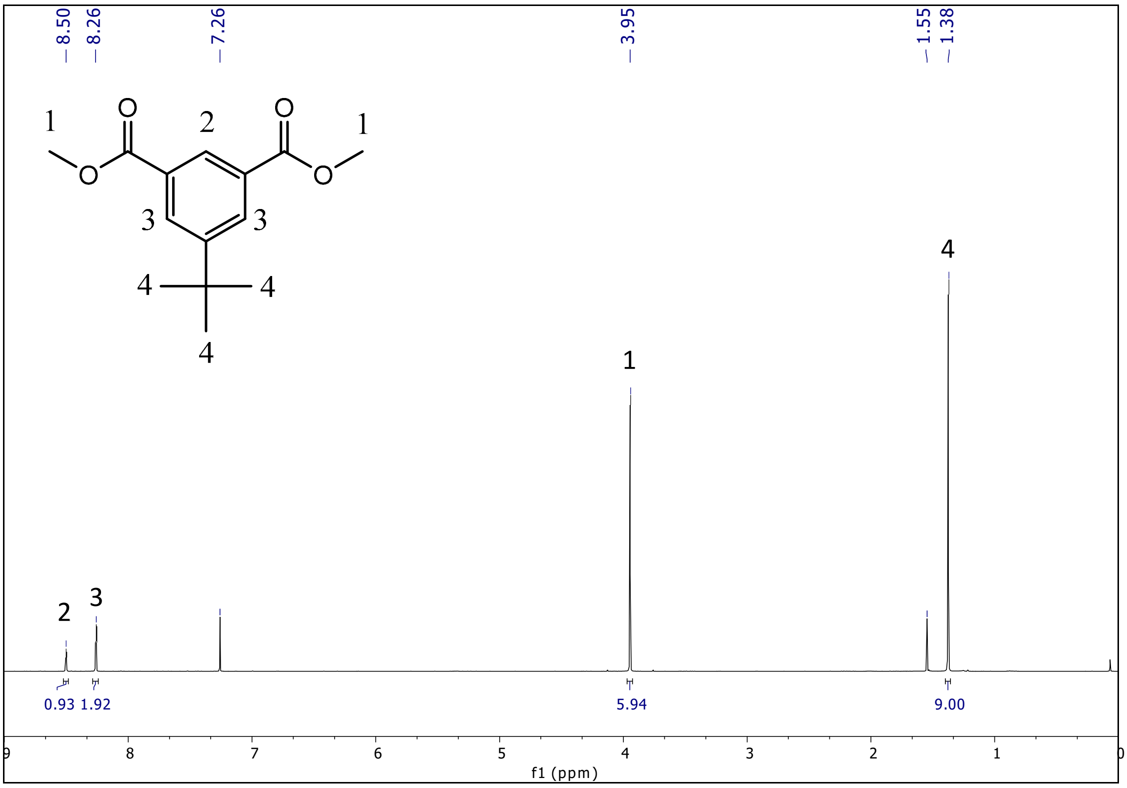

Figure S1. ^{1}H-NMR spectrum of dimethyl-5-(tert-butyl)isophthalate **(1)**.

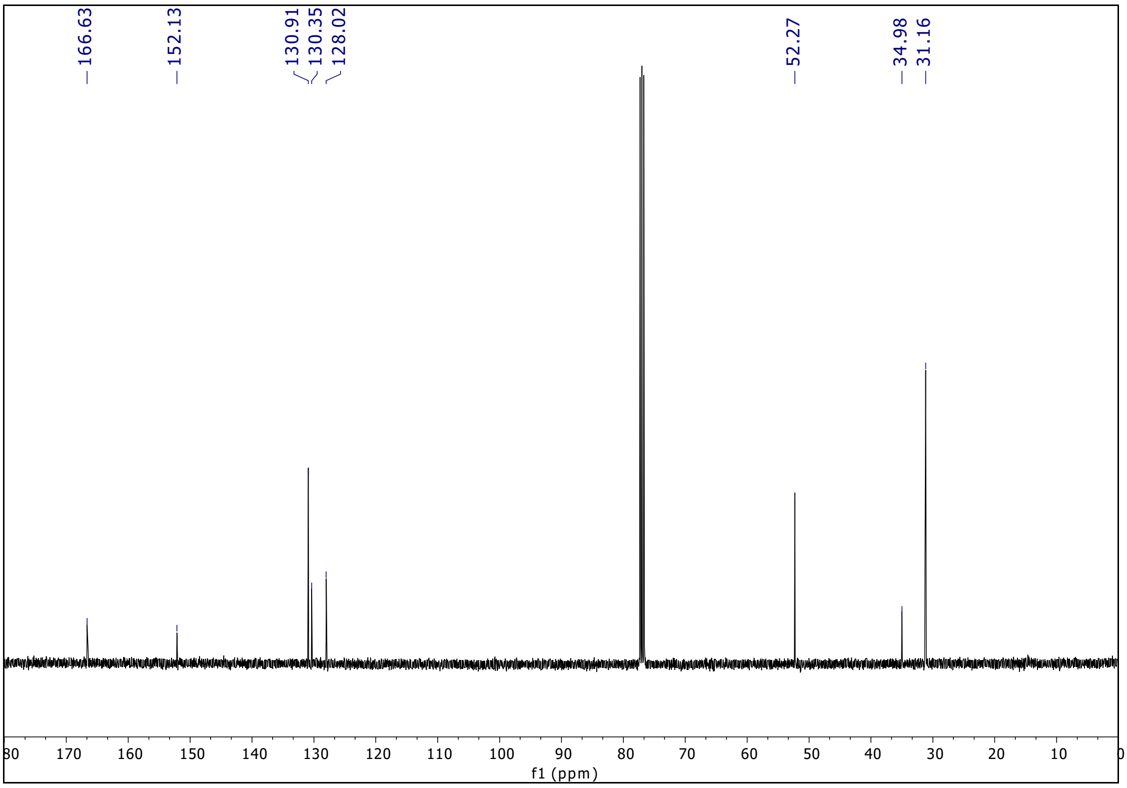

Figure S2. ^{13}C-NMR spectrum of dimethyl-5-(tert-butyl)isophthalate **(1)**.

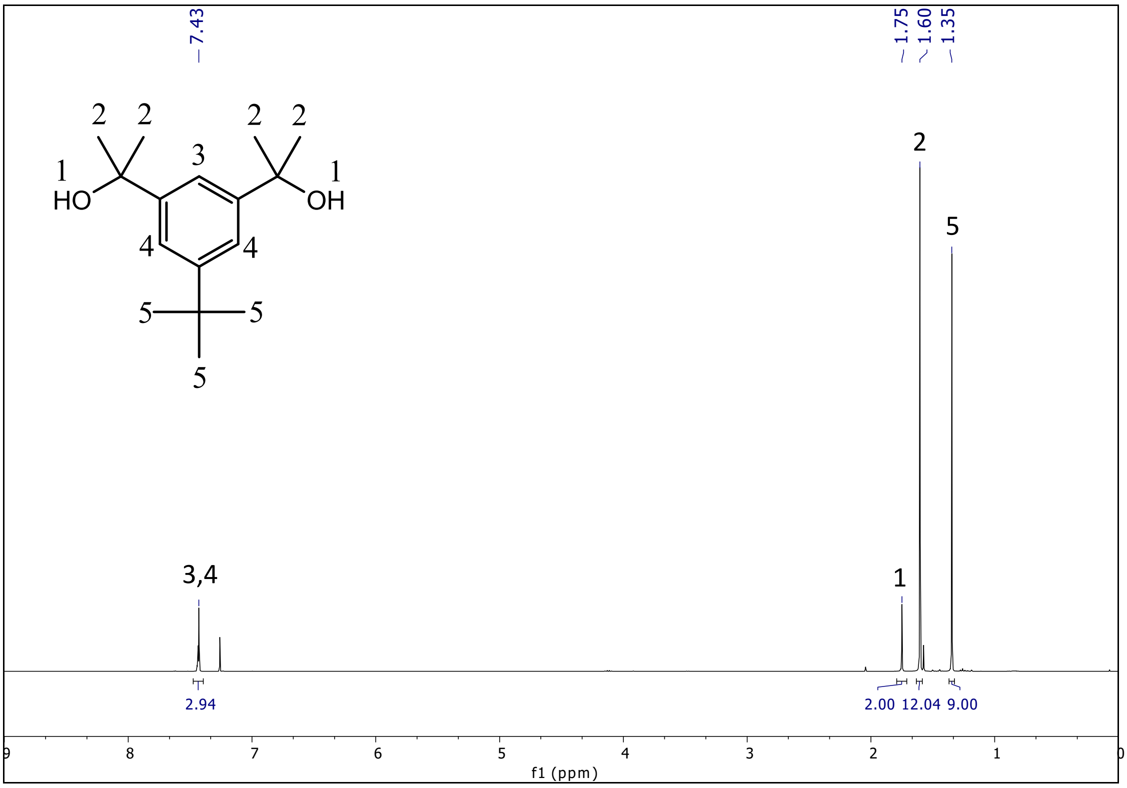

Figure S3. ^{1}H-NMR spectrum of 2,2'-(5-(tert-butyl)-1,3-phenylene)bis(propan-2-ol) **(2)**.

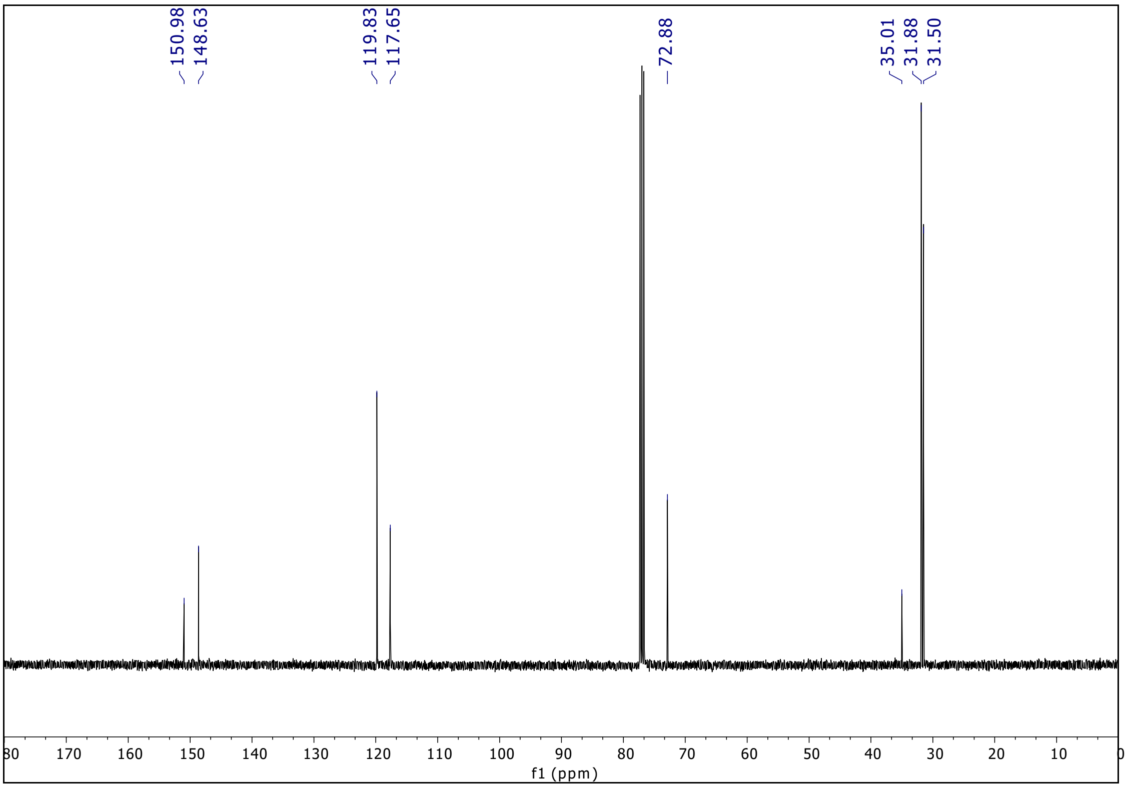

Figure S4. ^{13}C-NMR spectrum of 2,2'-(5-(tert-butyl)-1,3-phenylene)bis(propan-2-ol) **(2)**.

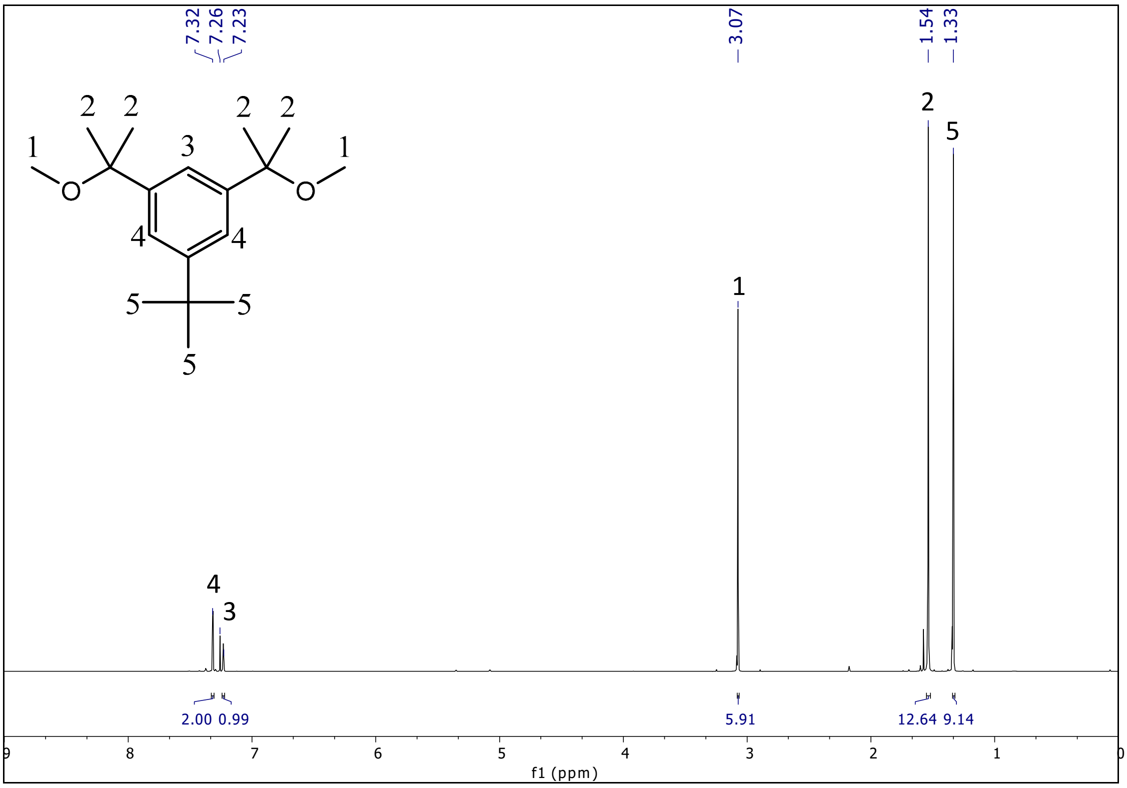

Figure S5. ^{1}H-NMR spectrum of 1-(tert-butyl)-3,5-bis(2-methoxypropan-2-yl)benzene **(3)**.

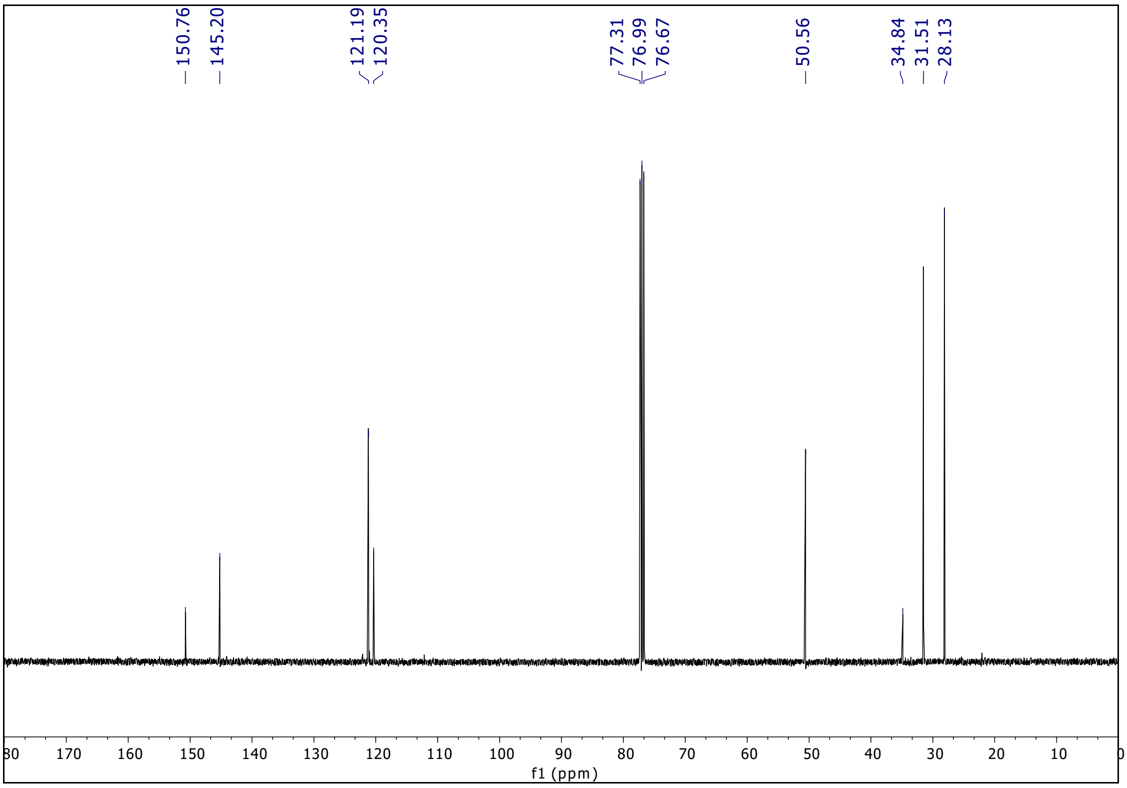

Figure S6. ^{13}C-NMR spectrum of 1-(tert-butyl)-3,5-bis(2-methoxypropan-2-yl)benzene **(3)**.

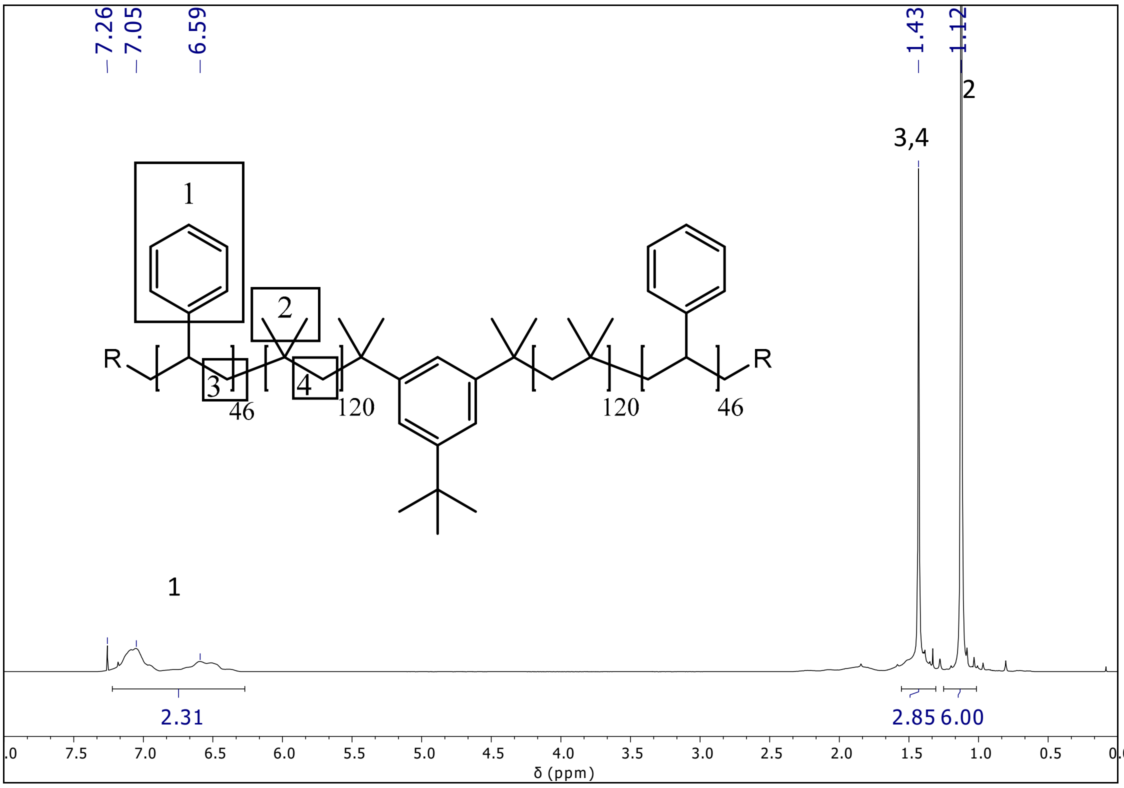

Figure S7. ^{1}H-NMR spectrum of SIBS-B4.

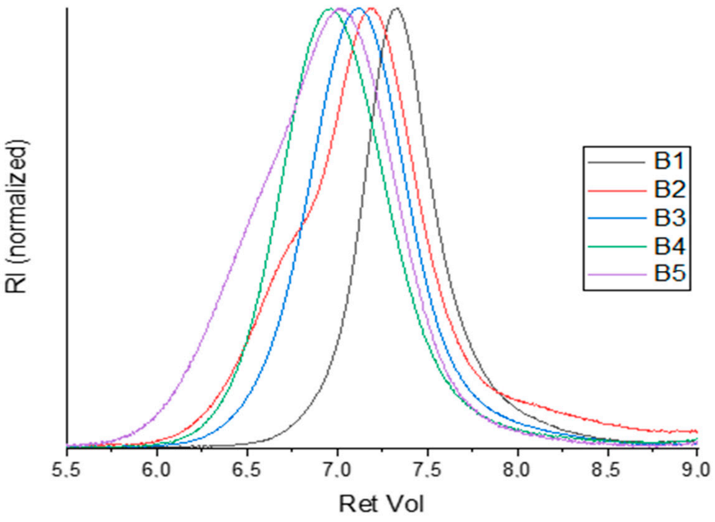

Figure S8. GPC graphs of all synthesized SIBS-polymers.

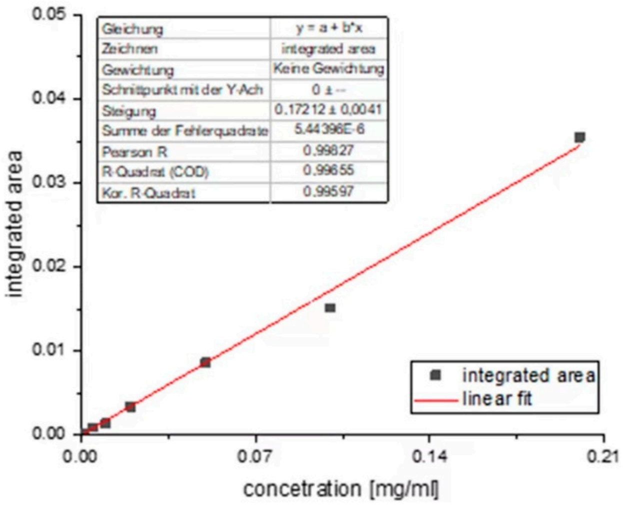

Figure S9. HPLC calibration for TA from 0.001 mg/ml to 0.2 mg/ml.

1. Gyor, M., et al., Living Carbocationic Polymerization of Isobutylene with Blocked Bifunctional Initiators in the Presence of Di-Tert-Butylpyridine as a Proton Trap. *Journal of Macromolecular Science-Pure and Applied Chemistry* **1992,** *29* (8), 639-653.
2. Orszagh, I., et al., Living carbocationic copolymerizations. I. Synthesis and characterization of isobutylene/p-methylstyrene copolymers. **1995,** *8* (4), 258-272.
